# Antinociceptive action of oxytocin involves inhibition of potassium channel currents in lamina II neurons of the rat spinal cord

**DOI:** 10.1186/1744-8069-5-63

**Published:** 2009-11-12

**Authors:** Jean Didier Breton, Pierrick Poisbeau, Pascal Darbon

**Affiliations:** 1 Nociception and Pain Department, Institut des Neurosciences Cellulaires et Intégratives, Unité propre de recherche 3212 du Centre National de la Recherche Scientifique, Université de Strasbourg, Strasbourg, France

## Abstract

**Background:**

Growing evidence in the literature shows that oxytocin (OT) has a strong spinal anti-nociceptive action. Oxytocinergic axons originating from a subpopulation of paraventricular hypothalamic neurons establish synaptic contacts with lamina II interneurons but little is known about the functional role of OT with respect to neuronal firing and excitability.

**Results:**

Using the patch-clamp technique, we have recorded lamina II interneurons in acute transverse lumbar spinal cord slices of rats (15 to 30 days old) and analyzed the OT effects on action potential firing ability. In the current clamp mode, we found that bath application of a selective OT-receptor agonist (TGOT) reduced firing in the majority of lamina II interneurons exhibiting a bursting firing profile, but never in those exhibiting a single spike discharge upon depolarization. Interestingly, OT-induced reduction in spike frequency and increase of firing threshold were often observed, leading to a conversion of the firing profile from repetitive and delayed profiles into phasic ones and sometimes further into single spike profile. The observed effects following OT-receptor activation were completely abolished when the OT-receptor agonist was co-applied with a selective OT-receptor antagonist. In current and voltage clamp modes, we show that these changes in firing are strongly controlled by voltage-gated potassium currents. More precisely, transient I_A _currents and delayed-rectifier currents were reduced in amplitude and transient I_A _current was predominantly inactivated after OT bath application.

**Conclusion:**

This effect of OT on the firing profile of lamina II neurons is in good agreement with the antinociceptive and analgesic properties of OT described *in vivo*.

## Background

Oxytocin (OT), a peptide hormone well known for its role in female parturition and lactation [1], has received recently a considerable attention for its implication in the regulation of complex brain functions [2] in both male and female. OT is synthesized and secreted by a subpopulation of hypothalamic neurons [3]. From the paraventricular hypothalamic nucleus, OT-containing parvocellular neurons send direct axonal projections to various structures of the central nervous system (CNS) including the spinal cord [4-7]. In the spinal cord, oxytocinergic terminals are found mainly in the most superficial layers of the dorsal horn and in the autonomic nuclei [4,7]. These spinal target structures contain an important density of OT binding sites [8-11] and synapses have been described in electron microscopy [12]. The colocalization of oxytocinergic terminals and OT binding sites in the superficial layers of the spinal cord strongly suggest that oxytocinergic transmission could modulate nociceptive processing. In good agreement, OT secreted in the spinal cord after PVN stimulation or exogenously-applied by intrathecal infusion, displayed antinociceptive properties in naïve rodents [13-16] or during the development of inflammatory [17-19] or neuropathic pain [20,21]. To explain some of these antinociceptive properties, it has been recently demonstrated that stimulation of the PVN reduced the spinal integration of nociceptive afferent messages mediated by C and Aδ fibers [22] presumably by elevating the GABAergic inhibition in the superficial layers of the spinal cord [23] while reducing glutamatergic sensorispinal transmission [24].

Little is known regarding the mechanism of action of OT on neuronal firing and therefore neuronal excitability. In most CNS structures, OT is generally described as excitatory, but inhibitory effects are also often seen depending on the concentration used, brain developmental stages, neuronal cell types and hormonal status [25]. To illustrate this complexity, OT was shown recently to induce spontaneous action potentials together with a presynaptic increase in GABAergic inhibitory transmission in hypothalamic magnocellular neurons grown in organotypic culture [26]. In this particular situation, where OT did not change the resting membrane potential, low threshold spikes were triggered by the IPSP-mediated rebound depolarization and were not seen in the presence of T-type calcium channel inhibitors. In the spinal cord, OT induced membrane depolarization in somatic motoneurons [27] and sympathetic preganglionic neurons [28,29] by a cellular mechanism likely involving TTX-resistant Na^+ ^conductances.

To investigate the effect of OT on electrophysiological properties of spinal nociceptive neurons, we chose to record lamina II interneurons which play a crucial role in the integration and initial filtering of peripheral nociceptive messages [30-32]. The main objective of this study was to characterize the effects of OT on the firing properties of lamina II neuron and to examine the possible contribution of K^+ ^channels to this modulation. Although they received little attention so far in the case of OT action, K^+ ^currents in the spinal cord play a major role in the control of the neuronal firing and excitability [33-36] and have already been shown to control the firing properties of lamina II neurons [37,38]. Two main voltage-gated K^+ ^currents have been described in spinal cord dorsal horn: a tetraethylammonium (TEA)-sensitive delayed rectifier K^+ ^current (IKDR) [39] and a (TEA)-insensitive fast inactivating K^+ ^current (I_A_) [39-41]. Moreover it has been shown that these currents are involved in the reduction of excitability produced by anesthetics in superficial dorsal horn neurons [36,42]. We have focused our attention on A-type K^+ ^currents which could play a crucial role in spinal pain processing [43].

## Results

### Oxytocin reduces the firing ability of a subpopulation of lamina II neurons

In the current clamp mode, perfusion of the specific OT receptor agonist TGOT (1 μM, n = 21), for up to 15 minutes, had no significant effect on the resting membrane potential (RMP) of lamina II neurons. In the absence of any current injection, the mean RMP was -63.6 ± 1.7 mV in control and -60.8 ± 2.0 mV in TGOT (n = 21; Student's *t*-test, p > 0.05). In line with what is generally observed in the absence of stimulation *in vivo *(see for example [44]), we also found that lamina II neurons recorded from our *in vitro *slice preparation never displayed spontaneously occurring action potentials at their resting membrane potential. Since no obvious effects on the resting membrane potential were observed, we next checked whether OT receptor agonists could affect the firing profile of lamina II neurons.

**Figure 1:**
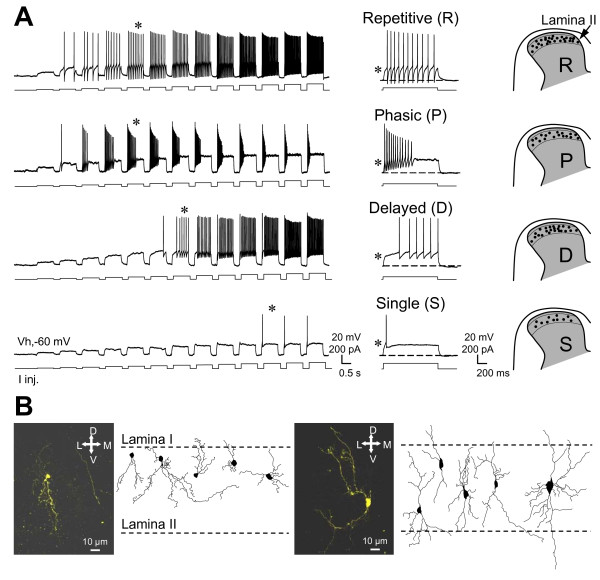
**Firing profiles of lamina II neurons recorded in rat spinal cord slices**. **A**: In the current clamp configuration of the whole-cell patch clamp, four distinct firing profiles were recorded in lamina II neurons in response to incremental depolarizing currents steps of 900 ms every 20 s. They are referred to as repetitive (R), phasic (P), delayed (D) and single spike (S). Typical repetitive firing profile is characterized by the tonic generation of action potentials during the depolarization. The phasic profile is characterized by a strong accommodation leading to a plateau. The delayed firing profile exhibits a time-dependent silent phase before bursting. Lastly, a small proportion of lamina II neurons displays only a single spike (sometimes two) at the beginning of the depolarization. Stars show characteristic firing profile at longer time-scale. Dashed lines represent -60 mV. As shown on the right diagram, no correlation could be found between the firing profile and the location of the recorded neuron. **B**: Biocytin revelation (images) and reconstruction (drawings) of recorded cells illustrating the large variety of morphology of lamina II neurons. In our hands, no correlation was observed between a specific morphology and the firing pattern.

As illustrated in figure 1A, we could easily identify the four main types of firing profile seen classically in the superficial layers of the spinal cord but sometimes in a different proportion. Using depolarizing current steps (see methods), we found here that lamina II neurons, recorded blindly, often displayed a repetitive ("R": 33.3%, n = 28/84 neurons, RMP = -59,4 ± 1.7 mV) or phasic ("P": 29.8%, n = 25/84 neurons, RMP = -59.3 ± 1.7 mV) firing profile whereas delayed firing ("D": 21.4%, n = 18/84 neurons, RMP = -60.9 ± 2.5 mV) and single spike ("S": 15.5%, n = 13/84 neurons, RMP = -60.5 ± 3.3 mV) were less frequent (Figure 1A).

**Figure 2:**
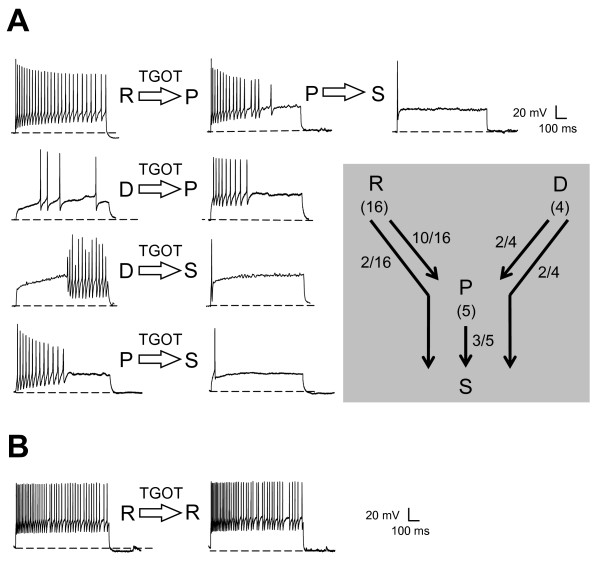
**Oxytocin reduces the firing ability of lamina II neurons**. **A**: Effects of TGOT (1 μM), a selective agonist of OT-receptor bath-applied for 15 minutes, on the different firing profiles expressed by lamina II neurons. Four different examples of firing profile conversion induced by TGOT. Diagram in the grey box gives the scheme of profile conversion observed after TGOT application. **B**: Example of a lamina II neuron exhibiting a repetitive profile that was not converted after a 15-min application of TGOT. Dashed lines represent -60 mV.

Bath application of the selective agonist TGOT (1 μM) induced a progressive reduction in the number of spikes emitted during a 900 ms current pulse in the majority of the recorded lamina II neurons (76%; n = 16/21; Figure 2). This change in firing ability was generally found stable and maximal after a minimal superfusion time of 15 minutes. A similar effect was obtained while using of the natural agonist OT (1 μM). In this case, bath-applied OT triggered a change in the firing pattern of 63% of the recorded neurons (n = 5/8). TGOT- and OT-mediated effects were both seen as a reduction in the number of action potentials generated during the 900 ms depolarizing current pulse of constant amplitude and, in 19 out of 29 neurons, as a progressive conversion of the firing profile (Figure 2A). The grey inset in Figure 2A summarizes the conversion scheme of lamina II neurons observed after superfusion of OT-receptor agonists.

We found that most of the neurons expressing a repetitive firing (n = 12/16) and all those with a delayed firing (n = 4/4) progressively showed a phasic profile. Out of these 16 OT-sensitive neurons, four were also further converted to a single spike profile. Moreover, neurons initially classified as phasic, were also sometimes converted into single spike profile (n = 3/5). It should be noted here that neurons exhibiting a single spike were unaffected by OT-receptor agonists application (n = 4). A subpopulation of "repetitive" lamina II neurons was also OT-insensitive (25%: 4/16 repetitive neurons; Figure 2A and 2B). We failed to reveal any change in the firing profile (e.g. no change in spike number and/or frequency) when OT was applied at lower concentration (10 nM, n = 8 and 100 nM, n = 8) and when OT (1 μM) or TGOT (1 μM) were co-applied with dOVT (1 μM, n = 6), a selective antagonist of OT receptor. Furthermore, dOVT (1 μM) alone did not affect the firing ability of the recorded neurons (n = 6). Lastly, the reduction in the firing ability induced by OT-receptor agonist was long-lasting since, even after a 1 hour washout, only a partial recovery could be observed.

After superfusion of OT-receptor agonists, the distribution of lamina II neurons based on their firing pattern was strongly modified. The majority of the neurons could now be classified as "single spike" (45%: n = 13/29) or "phasic" (41%: n = 12/29) whereas neurons with repetitive firing pattern represented only 14% (n = 4/29) of the total neuronal population instead of 41% in control condition (Chi-square test, p < 0.001). Neurons exhibiting a delayed action potential discharge were no longer seen after superfusion with OT-receptor agonists.

### Inhibition of A-type K^+ ^currents in lamina II neurons mimics the reduction of action potential firing ability induced by OT-receptor agonists

**Figure 3:**
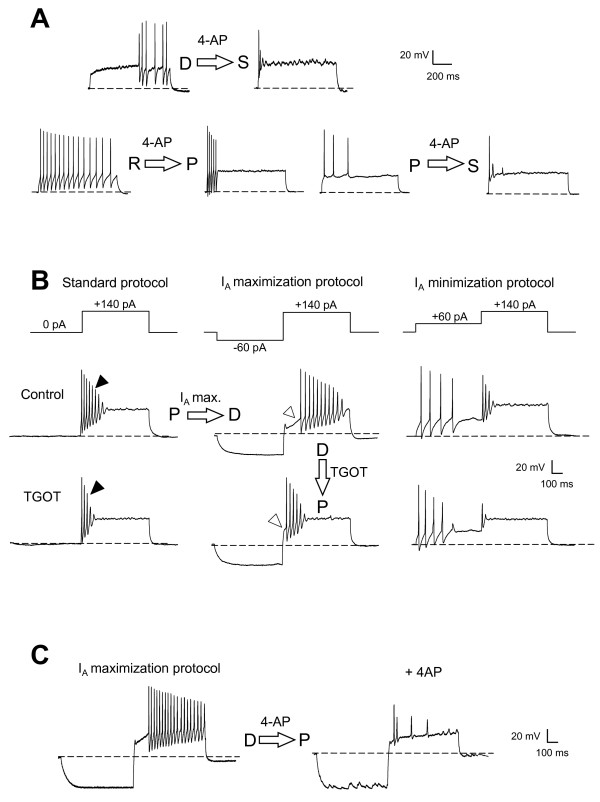
**Transient potassium current shapes the firing profile**. **A**: Modulation of the firing profile following the application of 4-AP, a blocker of transient K^+ ^currents (I_A_), in three different lamina II neurons. **B**: Firing profile changes exhibited by a phasic neuron (left panels, standard protocol) submitted to conditioning hyperpolarizing (middle panels) or depolarizing prepulse (right panels) aimed at maximizing or minimizing I_A _current, respectively. In these three conditions, the effect of TGOT application (bottom traces) is compared to the control situation (upper trace). Black arrowheads indicate the accommodation in spike frequency. White arrowheads show the delay time before obtaining the first action potential. **C**: Effect of 4-AP on a neuron exhibiting a delayed firing profile after a conditioning hyperpolarizing prepulse producing a large I_A _current. Dashed lines represent -60 mV.

In order to better understand how OT-receptor agonists could affect the firing profile of lamina II neurons, we focused our attention on the possible implication of potassium currents in this phenomenon. In a first step, we checked whether fast transient K^+ ^currents (A-type K^+ ^current: I_A_) could account for changes in the firing profiles observed in lamina II neurons. To do so, we recorded the action potential discharge in lamina II neurons, before and after the application of 4-aminopyridine (4-AP, 0.5 mM), a blocker of I_A_. As illustrated in Figure 3A, 4-AP mimicked the effects of TGOT (n = 5) but K^+ ^current inhibition was achieved within seconds after starting the superfusion. In delayed neurons, as expected, 4-AP suppressed the delay before the first spike and then converted them into phasic neurons. In repetitive neurons, 4-AP reduced the number of spikes emitted causing a conversion to phasic neurons. As expected, 4-AP also rapidly increased the initial firing frequency (at the beginning of the current pulse) by decreasing the interspike intervals. To summarize, the direct pharmacological blockade of I_A _mimicked the action of OT by reducing the number of emitted spikes and by eventually converting the firing profile (delayed into phasic, repetitive into phasic and phasic into single spike). However, OT did not reproduce the reduction of interspike interval, suggesting the involvement of an additional mechanism.

In addition to this preliminary experiment, we designed a protocol to manipulate the availability of I_A _channels (Figure 3B) in a separate set of neurons (n = 14; 7 repetitive, 5 phasic and 2 delayed profiles). To do so, a conditioning hyperpolarizing or depolarizing prepulse current injection was applied to the neurons in order to maximize or minimize the contribution of I_A _currents, respectively. As illustrated in figure 3B in the case of a "phasic" neuron, minimization of I_A _currents (I_A _minimization protocol, upper right) was sufficient to decrease the total number of spikes during the test pulse, thus mimicking the effect of OT. Interestingly, the maximization of I_A _currents (I_A _maximization protocol) converted the phasic firing pattern into a delayed one (n = 4/5 neurons) or increased even more the delay in one out of two delayed neurons. This procedure did not alter the repetitive pattern (n = 7). After I_A _current maximization, the delayed firing profile was suppressed after the application of TGOT since the delay to get the first action potential was no longer detectable (Figure 3B middle traces). Moreover, TGOT application induced a massive reduction in the number of emitted action potentials leading ultimately to a single spike profile (Figure 3B, right traces). As seen in figure 3C, the delayed firing profile resulting from the conditioning hyperpolarizing prepulse (I_A _maximization protocol) was also suppressed by 4-AP (0.5 mM, n = 2/2), similarly to what was seen after TGOT bath perfusion (Figure 3B). Taken together, these experiments indicated that A-type K^+ ^currents constituted an important functional target involved in the reduction of action potential firing ability induced by OT-receptor activation.

### OT modulation of potassium currents in lamina II neurons

To better understand how OT could modulate voltage-gated K^+ ^currents, we have recorded another set of lamina II neurons (n = 16) in the voltage-clamp mode and in the presence of TTX (0.5 μM). Under these conditions, we have been able to isolate the sustained (IKDR) and the transient (I_A_) component of total potassium currents (I_K_) in every lamina II neuron recorded (*see methods for K*^+ ^*current isolation procedures*). I_A _current was blocked by 4-AP (2 mM, n = 4, not shown), while TEA (10 mM) blocked IKDR currents (n = 4, not shown).

**Figure 4:**
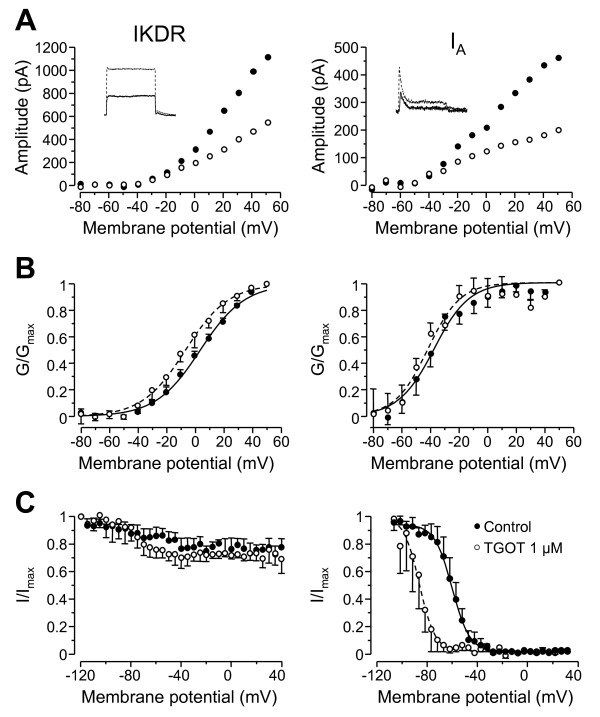
**Modulation induced by TGOT on properties of sustained (IKDR) and transient (I_A_) potassium currents**. **A**: Voltage/current relationship of the sustained (left graph) and transient K^+ ^currents (right graph) observed in a lamina II neuron in control (black circles) and after bath application of TGOT (1 μM, white circles). A typical current, obtained at a membrane potential of +50 mV, is shown in inset for both current components in control condition (black line) and after TGOT (dotted line). **B**, **C**: Activation (B) and inactivation curves (C) of sustained (left graphs) and transient components of K^+ ^currents (right graphs), respectively. Mean data are shown for 11 neurons, each recorded in the control situation (black circles and lines) and after application of 1 μM TGOT (white circles and dotted lines). All experiments were conducted in the presence of a steady-state concentration of TTX (0.5 μM). Values are expressed as means ± S.E.M.

After 15 minutes of bath application, we found that TGOT (1 μM) progressively reduced to a steady-state the peak amplitude of both sustained (IKDR: Figure 4A) and transient potassium currents (I_A_: Figure 4A) in 11 out of 16 lamina II neurons. This effect was never observed in the presence of dOVT (n = 6; not shown). The amplitude of IKDR, in response to a depolarization to +50 mV, was decreased by 42 ± 6.7% (n = 11; p < 0.001, Student's *t*-test) by TGOT (Figure 4A, left graph) but this effect was not accompanied by a change of the activation (Figure 4B, left) or inactivation properties (Figure 4C, left). In particular, the half-activation potentials (V_0.5_) were similar with mean values of 4.44 ± 1.4 mV in control and of -1.94 ± 2.22 mV after TGOT (n = 11; p > 0.05, Student's *t*-test). The slope factors (*k*) remained also unchanged (14.82 ± 0.72 mV in control and 15.17 ± 0.61 mV after TGOT). The amplitude of I_A _was also decreased after superfusion of TGOT (-44.2 ± 11.9%, n = 11; p < 0.01, Student's *t*-test) in response to a depolarization to +50 mV (Figure 4A, right graph). The parameters best describing the activation properties remained unaffected after application of TGOT (Control: V_0.5 _= -39.99 ± 4.31 mV, *k *= 12.27 ± 2.04 mV; TGOT: V_0.5 _= -40.90 ± 3.02 mV, *k *= 13.74 ± 4.31 mV; n = 11; p > 0.05, Student's *t *test; Figure 4B, right graph), whereas the inactivation curve (Figure 4C, right graph) was shifted toward more negative values in 4 out of 7 recorded neurons (control: V_0.5 _= -64.56 ± 3.13 mV vs. TGOT: V_0.5 _= -89.71 ± 5.57 mV; n = 4; p < 0.01, Student's t-test). The slope factor of the inactivation curve (Figure 4C, right graph) was, however, not significantly changed (control: *k *= 5.62 ± 1.99 mV vs. TGOT *k *= 3.90 ± 0.42 mV; n = 4, p > 0.05, Student's *t *test). The OT-associated shift of the inactivation curve toward a more negative value indicated that I_A _channels were inactivated at resting membrane potential values and therefore not available for spikes and interspike interval tuning.

## Discussion

Several studies are now supporting the idea that OT exerts a potent anti-nociceptive control after its release in the spinal cord from hypothalamo-hypophysal descending projections [14,15,45]. We have recently shown that this antinociceptive action is mediated, in part, by an increase in synaptic inhibition within the most superficial layers of the spinal cord [22,23], a mechanism which can also account for the previously described reduction of glutamatergic sensorispinal ascending messages [24]. In the present study, we now demonstrate that the effect of OT on synaptic transmission is accompanied by a modulation of potassium currents, which finely tunes the output properties of lamina II neurons receiving nociceptive information coming from peripheral nociceptive afferents. The observed reduction of A-type K^+ ^currents is particularly efficient in the firing profile coding. This mechanism is robust and compatible with a long-lasting antinociceptive action at the spinal level. Indeed pharmacological blockade or electrophysiological modulation of I_A _reproduced the main features of OT action on lamina II neurons.

### Action potential firing properties of lamina II neurons

As proposed by Prescott and De Koninck [46] for lamina I neurons, the firing profiles reflect differences in neuronal input-output functions. The diversity of input-output functions in lamina II neurons, which are the first relay in the nociceptive pathway, is therefore important determinant of the spinal pain processing. In this study, we found the four main classes of firing profiles (i.e. repetitive, phasic, delayed, single spike) already described in rat using various electrophysiological approaches [33,37,38,46-51]. Among these four firing profiles, repetitive and delayed firing neurons are both ideally suited to encode the intensity, the duration and the termination of afferent excitation. In contrast, phasic and single-spike neurons may predominantly operate as coincidence detectors [46], discharging only at the onset of multiple simultaneous afferent arrival inputs. Between studies, there is a large variability in the percentage of cells exhibiting the above described firing profiles, suggesting that the action potential discharge of lamina II interneurons, at least for a part of them, could be finely controlled by the activity of specific membrane voltage-gated ionic channels.

### OT-induced changes in lamina II neuronal firing

During our patch-clamp whole-cell experiments, we found that the recorded firing profiles were very stable over time (maximum 80 minutes) and this has also been shown in other reports [46,52]. While studying the effects of OT in the absence of synaptic activity, we never observed any significant changes of the resting membrane potential values while recording from lamina II neurons. With regards to our previously published results [23], only a very small fraction of lamina II neurons were capable of producing action potentials and this was the result of a rare OT-induced excitatory synaptic current summation. In contrast with these observations, a reduction in their firing ability was seen in the vast majority (> 70%) of the recorded neurons. The lack of effects of OT on membrane potential was also recently described for organotypic culture of magnocellular hypothalamic slices despite a major OT-induced increase in IPSPs frequency [26]. In line with this observation, OT did not induce any change in membrane current in superficial layer neurons grown in culture and recorded in the voltage-clamp mode [51]. Our findings are also in good agreement with the limited number of c-fos positive OT-activated lamina II neurons following a PVN stimulation [23]. We cannot exclude, however, that lamina I neurons might respond to OT by a depolarization and the emission of action potential. This was never the case while recording from lamina II neurons under our experimental conditions regardless of the OT-associated changes in excitatory and inhibitory synaptic controls [23].

In a large subpopulation of lamina II neurons, OT strongly and durably affected the action potential discharge. This effect, blocked by the selective OT-receptor antagonist dOVT, was seen as a progressive reduction of the number of emitted spikes and, in most cases, as a conversion of the initial firing profile. This phenomenon was also observed in a similar preparation after application of a μ opioid agonist [53]. Based on our experimental results, a preferential conversion scheme (Figure 2A, inset) can be proposed leading to a predominance of neurons with phasic and single spike firing after OT superfusion. Data obtained using unitary spinal cord neurons recording *in vivo *are already available in the literature to support this model [45,54], although little is known about the mechanisms involved. In the present study and as seen in figure 1A, lamina II neurons exhibiting a single spike had a high rheobase i.e. they required a higher current injection or more synaptic inputs to trigger their first and single spike. We can conclude here that these OT-sensitive lamina II neurons are becoming less excitable in comparison with "Repetitive", "Phasic" or "Delayed" cells which usually had a lower rheobase. The proportion changes among the different firing profiles should also modify the processing of nociceptive information. Indeed, OT reduced the number of integrators (repetitive/delayed) and increased the number of coincidence detectors (phasic/single spike) which are less prone to encode intensity of the stimulus according to Prescott and De Koninck [46].

The inhibitory or excitatory nature of lamina II interneurons has also to be taken into account while analyzing OT-induced changes in excitability. Previous studies have attempted to establish a correlation between firing discharge profile and the neurochemical nature of lamina II interneurons. Repetitive firing seems to be mainly expressed by islet cells [55], which are very likely to be inhibitory [55-58]. In our hands, OT-induced reduction of excitability is affecting a large population of these neurons (75%). Phasic firing patterns have been reported for either excitatory interneurons [57] or inhibitory interneurons [56] and it is difficult to predict the consequence of OT action without knowing their precise connectivity in the network. Delayed firing neurons are all OT-sensitive. They are likely to be excitatory interneurons and possibly serve as output towards lamina I projecting neurons [57]. If true, the OT-induced decrease in neuronal excitability will then reinforce the filter function of lamina II while processing peripheral afferent nociceptive messages.

Together, OT-receptor activation is likely to alter the processing of the nociceptive signal by lamina II neuronal network before its forwarding to supraspinal centers. Establishing the relative contribution of this mechanism in comparison to others previously described, that also affect synaptic transmission onto lamina II neurons [23,24], will require further investigation in order to better understand how OT produces its antinociceptive action.

### Lamina II neuron firing and OT modulation of K^+ ^currents

The major K^+ ^currents involved in the control of action potential firing of superficial dorsal horn neurons are a TEA-sensitive delayed rectifier K^+ ^current, IKDR [39], and a TEA-insensitive fast inactivating K^+ ^current, I_A _[39-41]. Studies analyzing the action of anesthetics at the superficial spinal level have shown that reduction of potassium currents could lead to excitability reduction [36,42]. Any modulation of these currents could therefore modify the firing profile and the excitability of lamina II neurons. Our experiments showed that a reduction of I_A_, in addition to the expected suppression of the delay before the first spike, induced a reduction of the number of emitted spikes as well as an augmentation of the firing frequency. This is in full agreement with the known action of I_A_. Moreover, we confirmed that OT conversion of the firing profiles was actually involving the modulation of A-type K^+ ^current by (i) minimizing/maximizing I_A _currents (Figure 3B), (ii) mimicking the effect of OT with the I_A _blocker, 4-AP (Figure 3A), and (iii) showing that OT reduced the I_A _current amplitude and shifted the inactivation curve to more negative (hyperpolarized) values in voltage-clamp mode (Figure 4A, C). This suggests that I_A _is progressively unable to be reactivated after the first spike as shown with 4-AP (Figure 3A). However, I_A _blockade induced an expected decrease in interspike interval which is not seen with OT. We have to keep in mind, however, that OT acts on both K^+ ^currents. Indeed, Olschewski et al. [36] have shown that a reduction of IKDR increased the interspike intervals duration in lamina II neurons via a stronger inactivation of voltage-gated Na^+^channels during prolonged action potential, leading to an increased of the time needed for the channel to recover from inactivation. In summary, OT action on both K^+ ^currents has multiple consequences: a decrease in firing frequency due to a reduction of IKDR concomitant with a reduction in the number of emitted spikes leading to a firing profile conversion, from repetitive profile to phasic profile to single spike profile.

Lastly, we have noticed that OT action persisted long after its washout. During our whole-cell patch-clamp experiments, we were only able to get a partial recovery after 1 hour of washout. This long lasting effect of OT on spinal neuronal excitability is in good agreement with the specific reduction in C/Aδ-associated discharge of superficial layer neurons obtained with extracellular unit recording after PVN stimulation or topical application of OT in anesthetized rats [45]. This observation suggests that OT-receptor activation may stimulate intracellular amplification cascades involving enzymatic processes, which are difficult to stop or reverse. ERK 1/2 might be involved in this process because it was shown to exert an inhibitory regulation on transient I_A _currents in dorsal horn neurons [34]. This ERK pathway may also result from the convergence of PKA and PKC pathways, leading to a reduction of neuronal excitability [33,34] and could be recruited by OT-receptor activation, which uses the same intracellular pathway [59].

## Conclusion

In summary, we show here that the reduction of the firing ability induced by OT in lamina II neurons is associated with a reduction in transient (I_A_) and delayed (IKDR) K^+ ^currents. With regards to our previous study, we can propose that OT anti-nociceptive action is, at least, mediated by an increased inhibitory synaptic tone controlled by presynaptic OT-receptors [23] and a reduced neuronal firing ability involving an important OT-receptor-dependent inhibition of K^+ ^currents (this study). With regards to the interesting properties of OT in the spinal nociceptive system, it will be of fundamental importance to identify the intracellular partners recruited after OT-receptor activation in order to target this endogenous pain modulation pathway for therapeutical purpose.

## Methods

### Ethical approval

All experiments were conducted in accordance with the directive set by the European Community Council Directive (86/69/EEC, November 24, 1986) and authorization of the French Department of Agriculture (License no. 67-116 to PP).

### Spinal cord slice preparation

In this study we used 15 to 30 day-old Wistar rats (Depré, St Doulchard, France) anesthetized with a single i.p. injection of ketamine (150 mg/kg, Imalgène™, Meriel, France). Dissection procedure was described in detail previously [60]. Briefly, the spinal cord was removed by hydraulic extrusion and placed into ice-cold sucrose artificial cerebrospinal fluid (ACSF) containing (in mM): 248 sucrose, 11 glucose, 23 NaHCO_3_, 2 KCl, 1.25 KH_2_PO_4_, 2 CaCl_2_, 2 MgSO_4 _continuously bubbled with 95% O_2_-5% CO_2_. Transverse slices (600 μm thick) were cut from the lumbar segment of spinal cord using a Leica fresh tissue slicer (V1000S) and stored at room temperature into regular ACSF (in mM): 126 NaCl, 26 NaHCO_3_, 1.25 NaH_2_PO_4_, 2.5 KCl, 2 CaCl_2_, 2 MgCl_2_, 10 Glucose, pH = 7.35, continuously bubbled with 95% O_2_-5% CO_2_. Spinal cord slices were incubated at least 1 hour before recording. During the recordings, slices were continuously superfused with oxygenated ACSF at a mean flow-rate of a 3 ml per minute.

### Electrophysiological recordings, data acquisition

All electrophysiological experiments were performed at room temperature. Lamina II neurons were recorded blindly in the whole-cell configuration of the patch-clamp technique and filled with biocytin (see composition of the intracellular solutions below) in order to confirm their general morphology and their location within lamina II (Figure 1B).

Fast current-clamp and voltage-clamp recordings were obtained using an Axopatch 200B amplifier (Axon Instruments, USA). Electrophysiological recordings were sampled at 13 kHz in current-clamp and 10 kHz in voltage-clamp mode, respectively, then low-pass filtered at 5 kHz, digitized, and stored using pClamp 6.0 software (Axon Instruments, USA). Patch pipettes were made from borosilicate glass capillaries (Clark Electromedical Instruments, UK) using a horizontal laser puller (P-2000; Sutter Instruments, USA). The electrode resistance was 3.5-4.5 MΩ. The patch pipettes were filled with an intracellular solution containing (in mM): 80 K_2_SO_4_, 8 KCl, 2 MgCl_2_, 10 HEPES, 1 Mg-ATP, 10 Biocytin, pH 7.3 (adjusted with KOH). Capacitance transients, series resistance were compensated electronically. In our experimental conditions, cell dialysis was without effect on the firing properties exhibited by lamina II neurons, sometimes recorded for more than an hour. During the pharmacological experiments, we have recorded only one neuron per spinal cord slice.

### Experimental protocols and analysis

In current-clamp mode, the membrane potential was adjusted at -60 mV. Firing profiles were then characterized (Figure 1A, left) using current step injections (from -80 to 200 pA, 10 pA increments for 900 ms, repeated every 20 seconds). This characterization was performed 5-10 minutes after the beginning of the recording in order to ensure stable recordings and optimal dialysis of the intracellular compartment. To investigate the possible role of washout on cell firing properties, some neurons were recorded for more than an hour (n = 7). No significant changes in the firing profiles (spike frequency and spike amplitude), passive membrane properties and resting membrane potential were noted indicating that the intracellular dialysis with our experimental intracellular solution had no effect on the passive and active membrane properties of lamina II neurons (not shown). As seen in figure 1A, we found the four main firing profiles (Figure 1A) which have been described previously [38,47,49]. We were unable to reveal a positive correlation between location in lamina II (Figure 1A, right) or morphology and firing profile (Figure 1B). Neurons were kept for analysis only if they displayed stable resting membrane potentials more negative than -45 mV, proper spike overshoots (> 15 mV) and series resistance below 20 MΩ along the experiment.

In voltage-clamp mode, the membrane was held at -60 mV to study voltage-dependent potassium currents. Sodium currents were blocked by the addition of tetrodotoxin (0.5 μM TTX, Latoxan, France). Capacitive transient and leak conductance were subtracted using the P/4 protocol built-in function of pClamp software. Two different potassium current families could be recorded in lamina II neurons. The sustained outward potassium current (IKDR) blocked by tetraethylammonium (TEA, 10 mM; Fluka, Switzerland) and the transient outward potassium current (I_A_) blocked by 4-aminopyridine (4-AP, 2 mM; Sigma, France). Alternatively, these potassium currents could be separated on the basis of their electrophysiological properties. The total potassium current was obtained from a holding potential of -60 mV; through a series of voltage steps (from -100 to 50 mV, duration of 250 ms every 5 s) preceded by a conditioning step to -100 mV (250 ms). To record IKDR, the conditioning step was set to -40 mV to inactivate I_A _current. I_A _was calculated by a trace by trace subtraction between the two previous protocols. IKDR amplitude was measured at steady state, at the end of the step. I_A _amplitude was measured has the difference between the peak amplitude value and the steady state value. Activation and inactivation properties of both currents were obtained using a variable conditioning pulse (from -120 to +40 mV, steps of 250 ms) before a test pulse to 0 mV (250 ms). Activation and inactivation curves were well fitted with a Boltzmann function: y = 1/[1 + e^(V0.5-V)/*k*^] and y = 1/[1 + e^(V-V0.5)/*k*^], respectively; where V_0.5 _is the voltage of half-maximal activation, V is the holding membrane potential and *k *is the slope factor.

All recorded cells were analyzed using the pClamp 9.0.2 (Axon Instrument, USA). Quantitative results are expressed as mean ± S.E.M. Statistical analysis was performed using the parametric one-way ANOVA with Student's *t*-tests to compare means as indicated in the result section or chi-squared test. Statistical significance was considered to be reached for p < 0.05.

### Drug application and perfusion solution

We used different ligands of OT-receptor: natural oxytocin (OT), selective OT-receptor agonist [Thr^4^, Gly^7^]-oxytocin (TGOT; both from Sigma, France) and selective OT-receptor antagonist d(CH_2_)_5_- [Tyr(Me)^2^, Thr^4^, Orn^8^, des-Gly-NH_2 _^9^]-vasotocin (dOVT; Bachem, Germany). These drugs were bath-applied at their final concentration for a minimal time period of 15 minutes. Drugs were prepared as ×1000 stock solutions in distilled water, except OT, TGOT and dOVT that were dissolved in a molar acetic acid solution 0.25% (v/v) in distilled water. This vehicle had no effects on the membrane voltage, neuronal firing properties or K^+ ^current kinetics in the present study.

## Competing interests

The authors declare that they have no competing interests.

## Authors' contributions

JDB carried out the experiments, analyzed the data and revised the manuscript. PP conceived the project and supervised the study. PD analyzed the data, drafted and revised the manuscript. All authors read and approved the final manuscript.
